# Mechanical Ventilation Impairs IL-17 Cytokine Family Expression in Ventilator-Associated Pneumonia

**DOI:** 10.3390/ijms20205072

**Published:** 2019-10-12

**Authors:** Fien H. R. De Winter, Bart ’s Jongers, Kenny Bielen, Domenico Mancuso, Leen Timbermont, Christine Lammens, Vincent Van averbeke, Jan Boddaert, Omar Ali, Jan Kluytmans, Alexey Ruzin, Surbhi Malhotra-Kumar, Philippe G. Jorens, Herman Goossens, Samir Kumar-Singh

**Affiliations:** 1 Molecular Pathology Group, Laboratory of Cell Biology and Histology, Faculty of Medicine and Health Sciences, University of Antwerp, Universiteitsplein 1, B-2610 Wilrijk, Belgium; 2 Laboratory of Medical Microbiology, Vaccine and Infectious Disease Institute, University of Antwerp, Universiteitsplein 1, B-2610 Wilrijk, Belgium; 3 Microbial Sciences, R&D BioPharmaceuticals, AstraZeneca, Gaithersburg, MD 20877, USA; 4 Julius Center for Health Sciences and Primary Care, University Medical Center Utrecht, HP Stratenum 6.131, PO Box 85500, 3508 GA Utrecht, The Netherlands; 5 Department of Critical Care Medicine, Antwerp University Hospital and University of Antwerp, LEMP, Wilrijkstraat 10, B-2650 Edegem, Belgium

**Keywords:** mechanical ventilation, ventilator-associated pneumonia, VAP, *Pseudomonas aeruginosa*, IL-17, IL-17A, IL-22, IFNγ

## Abstract

Mechanical ventilation (MV) is the primary risk factor for the development of ventilator-associated pneumonia (VAP). Besides inducing a pro-inflammatory T-helper (Th)-1 cytokine response, MV also induces an anti-inflammatory Th2 cytokine response, marked by increased IL-4 secretion and reduced bacterial phagocytic capacity of rodent lung macrophages. Since IL-4 is known to downregulate both Th1 and Th17 cytokines, the latter is important in mediating mucosal immunity and combating bacterial and fungal growth, we studied and showed here in a rat model of MV that Th17 cytokines (IL-17A, IL-17F, and IL-22) were significantly upregulated in the lung as a response to different MV strategies currently utilized in clinic. To study whether the increased IL-4 levels are associated with downregulation of the anti-bacterial Th17 cytokines, we subsequently challenged mechanically ventilated rats with an intratracheal inoculation of *Pseudomonas aeruginosa* (VAP model) and showed a dramatic downregulation of IL-17A, IL-17F, and IL-22, compared to animals receiving the same bacterial burden without MV. For the studied Th1 cytokines (IFNγ, TNFα, IL-6, and IL-1β), only IFNγ showed a significant decrease as a consequence of bacterial infection in mechanically ventilated rats. We further studied IL-17A, the most studied IL-17 family member, in intensive care unit (ICU) pneumonia patients and showed that VAP patients had significantly lower levels of IL-17A in the endotracheal aspirate compared to patients entering ICU with pre-existing pneumonia. These translational data, obtained both in animal models and in humans, suggest that a deficient anti-bacterial Th17 response in the lung during MV is associated with VAP development.

## 1. Introduction

Patients receiving mechanical ventilation (MV) for more than 48 h have an approximately 10-fold higher likelihood of developing pneumonia compared to non-ventilated patients in intensive care units (ICUs) [1,2]. MV causes tidal volume (V_T_)-dependent ventilator-induced lung inflammation. This inflammation is characterized by both activation of proinflammatory T_H_1 cytokines such as tumor necrosis factor alpha (TNF-α), interleukin 1β (IL-1β), interferon gamma (IFN-γ), and interleukin 6 (IL-6), as well as lung infiltration of innate cells such as neutrophils and macrophages [3,4,5,6,7,8]. We recently showed that even using a so-called “lung-protective” ventilation strategy in rats induces these T_H_1 cytokines, and at the same time it also induces an anti-inflammatory T_H_2 type response marked by elevated IL-4 secretion [7]. Interestingly, while infiltration of innate cells, such as neutrophils and macrophages, was also noted, macrophages isolated from bronchoalveolar lavage (BAL) were specifically activated towards anti-inflammatory M2 macrophages, displaying reduced bacterial phagocytic capacity [7]. Mechanically ventilated rats, when challenged with bacterial lung inoculation to mimic a ventilator-associated pneumonia (VAP) model, had a higher lung bacterial burden compared to non-mechanically ventilated animals receiving the same bacterial dose [7,9]. Interestingly, blocking of the IL-4 signaling normalized the lung bacterial burden in the VAP-induced animals [7]. Thus, while it appears that T_H_2 cytokines could be important in transient immunosuppression in ventilated lungs favoring bacterial and fungal growth, the precise downstream mechanisms remain unknown.

The propagation and regulation of an immune response is driven by a network of regulatory T and effector cells as well as their innate immune counterparts. The interplay between different effector T cells determines the direction of the immune response towards inflammation or its resolution. For instance, T_H_2 cells and its main cytokine IL-4 are crucial in maintaining the balance between proinflammatory and anti-inflammatory pathways [10]. Similar to the proinflammatory T_H_1 cytokines, T_H_17 cytokines such as IL-17A, IL-17F, and IL-22 have a major impact on epithelial cells in various tissues and are important in mediating mucosal immunity, particularly against Gram-negative bacteria and fungal infections, by inducing neutrophil chemotaxis [11,12,13,14].

However, published data on IL-17/IL-22 in MV or VAP are conflicting. One study on ventilated patients showed that T_H_17 cells were increased both in the peripheral blood and in the lung in VAP patients compared to ventilated patients who did not develop pneumonia [15]. In contrast, another study on ventilated patients showed a reduced number of T_H_17 cells in lung lavage fluid of VAP patients compared to those who did not develop VAP, suggesting that T_H_17 cells were protective against VAP [16]. However, IL-17 levels in BAL fluid showed an opposite trend, where IL-17 levels were higher in VAP than non-VAP patients, though it should be noted that IL-17 levels in most of the patients were below the limit of detection of the assay [16]. Similarly, although IL-17 in VAP animal models has not been studied, mice ventilated for one hour with V_T_ 7 mL/kg were reported to have increased IL-22 but decreased IL-17 levels [17].

Here, we aimed to clarify the expression pattern of IL-17-related cytokines as a response to MV and VAP in both rodent models and patients and further questioned whether bacterial-induced stimulation of T_H_1 and T_H_17 cytokines could be dampened due to MV.

## 2. Results

### 2.1. Mechanical Ventilation Induces V_T_ Dose-Dependent T_H_17 Cytokine Expression in Lungs of Rats

**Figure 1:**
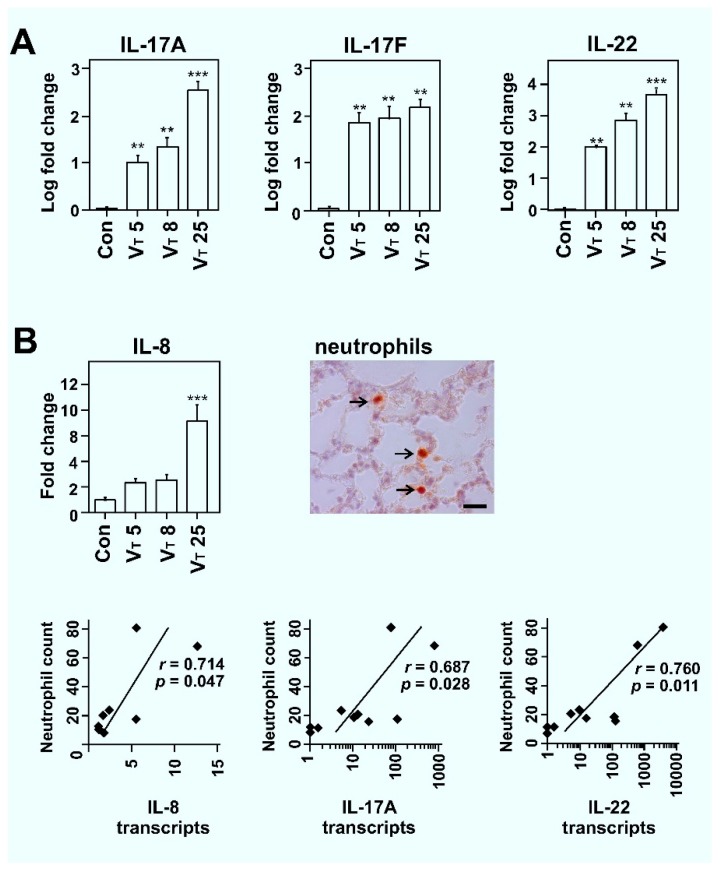
Effect of mechanical ventilation on transcript levels of *IL-17A*, *IL-17F*, *IL-22*, and *IL-8* and correlations with tissue neutrophils. (**A**) *IL-17A*, *IL-17F*, and *IL-22* lung transcripts analyses of spontaneously breathing control (Con), and in tidal volume (V_T_) 5, V_T_ 8, and V_T_ 25 mL/kg ventilation groups of Wistar rats analyzed after 2 h of mechanical ventilation (MV). Data is presented as averages ± standard error of mean (SEM); *p < 0.05, **p < 0.01; and ***p < 0.001; asterisk above the error bars denotes significance against spontaneously breathing control group; *n* = 6 animals per group. (**B**) A similar *IL-8* lung transcript analysis and Spearman correlation between lung neutrophil counts (neutrophils were detected with an anti-neutrophil antibody; scale bar, 20 μm; arrow indicates neutrophils) and transcript levels of *IL-8, IL-17A*, and *IL-22* (*n* = 10 animals per group).

To study whether T_H_17 cytokine expression is induced by MV, we ventilated rats for 2 h using three different lung-ventilation strategies: (i) A lung protective ventilation setting (avoiding injury by stretching) of V_T_ 5 mL/kg commonly employed in both healthy patients and those suffering from severe forms of lung injury to prevent further damage [18]; (ii) a V_T_ 8 mL/kg ventilation protocol, the highest level considered more or less “not damaging” to lungs by overstretching [6,19]; and (iii) a V_T_ 25 mL/kg ventilation protocol, which causes lung injury and neutrophil influx by itself, and therefore commonly employed to establish acute severe lung injury-like features in rodents [20]. Studying lung transcript levels for T_H_17 associated cytokines, we observed that *IL-17A*, *IL-17F*, and *IL-22* transcripts were modestly but significantly elevated in all studied ventilation groups in a volume-dependent manner (*p* < 0.01, for all; Figure 1A).

We further studied the transcript levels of *IL-8*, a potent chemoattractant of neutrophil in lung injury and an essential component of the innate immune system acting as a first line of defense against invading microorganisms and fungi [21,22]. IL-17 can activate the neutrophil/T_H_17 cell-dependent immune response through different cytokines and chemokines that includes IL-8 [14]. Recent data also suggest that neutrophil production by IL-17 enhances host antibacterial ability [23], and both IL-17 and neutrophils unexpectedly contribute to type 2 immunity [24]. We show here that *IL-8* transcripts were non-significantly elevated for V_T_ 5 and V_T_ 8 groups and significantly (approximately nine-fold; *p* < 0.001) elevated for the V_T_ 25 group (Figure 1B). Furthermore, we studied whether levels of IL-17-related cytokines, or neutrophil chemotactic cytokine IL-8, correlated with neutrophil infiltration in ventilated lungs. For this, a subset of samples was stained using a neutrophil-specific antibody and their numbers estimated as described [7,25]. We previously showed that mechanical ventilation in rats causes a V_T_-dependent increase in interstitial neutrophils (*p* < 0.01) [7]. We further show here that lung neutrophils in ventilated lungs correlated well with transcript levels of *IL-8* (*r* = 0.714, *p* = 0.047), *IL-17A* (*r* = 0.687, *p* = 0.028), and *IL-22* (*r* = 0.760, *p* = 0.011). These data suggest that increased expression of T_H_17 cytokines correlated with neutrophil infiltration in ventilated lungs through, or together with, IL-8 (Figure 1B).

### 2.2. Dampened IL-17 Response after Infection in Mechanically Ventilated Rats

We previously showed that MV induces besides a T_H_1 response [5,6,7], also a significant T_H_2 cytokine response [7], which is known to suppress T_H_1 and T_H_17 cytokine production [10]. We questioned whether this impacts T_H_17 and T_H_1 cytokine expression induced by bacterial infection. For this, we ventilated rats with V_T_ 8 mL/kg for 2 h followed by an intra-tracheal challenge with *Pseudomonas aeruginosa* (MV+P_A_ group) to mimic VAP. The levels of T_H_17 and T_H_1 cytokines of VAP animals were compared with animals that received the same bacterial dose without ventilation (P_A_ group). *P. aeruginosa* was used as an infectious organism as it is an important cause of hospital-acquired infections including VAP and it is known to cause a strong inflammatory response in lung epithelial cells [26,27,28].

**Figure 2:**
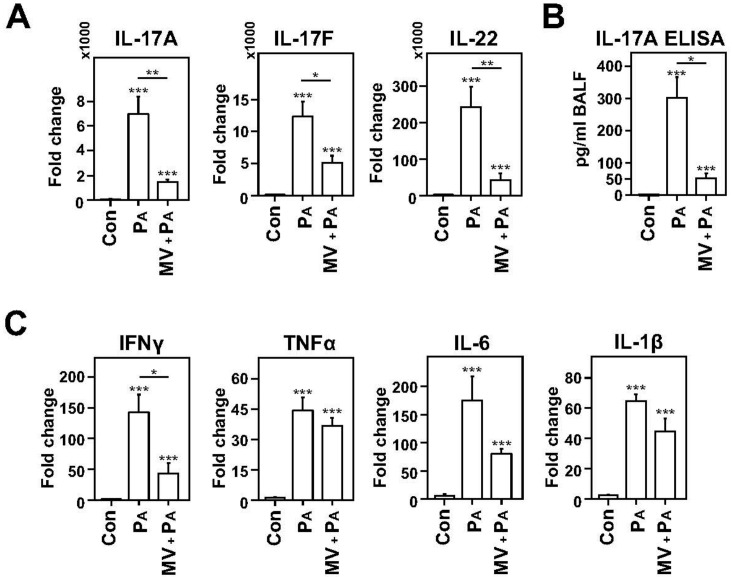
Dampening of proinflammatory T_H_17 and, to a limited extent, T_H_1 cytokines induced by infection in mechanically ventilated rats. (**A**) Lung transcript levels of *IL-17A*, *IL-17F*, and *IL-22* are reduced in mechanically ventilated animals infected with *Pseudomonas aeruginosa* (MV+P_A_) compared to non-ventilated, infected animals (P_A_) euthanized after 24 h. (**B**) IL-17A protein measured in bronchoalveolar lavage (BAL) fluid was also reduced in MV + P_A_ group compared to the P_A_ group. (**C**) Similarly, lung transcript levels of T_H_1 cytokines showed a declining trend for MV+P_A_ group compared to the P_A_ group, but these differences were significant only for *IFN-γ*. (**A**–**C**) Data are presented as averages ± SEM; * *p* < 0.05, ** *p* < 0.01; and *** *p* < 0.001; asterisk above the error bars denotes significance against control group; *n* = 5–6 animals per group.

Bacterial challenge with *P. aeruginosa* of lungs in non-ventilated rats induced an increase in expression of T_H_17 cytokines (*IL-17A*, *IL-17F*, and *IL-22*; *p* < 0.001 for all; Figure 2A). However, the same bacterial challenge in mechanically ventilated rats at V_T_ 8 mL/kg (MV+P_A_ group) caused significantly lower transcript levels of *IL-17A* (four-fold; *p* < 0.01), *IL-17F* (two-fold; *p* < 0.05), and of *IL-22* (four-fold; *p* < 0.01) when compared to the P_A_ group. We further confirmed the reduction in IL-17A at the protein level with an ELISA, where a six-fold decrease in protein expression in the MV+P_A_ (VAP) group was observed compared to the P_A_ group (*p* < 0.05; Figure 2B). Similarly, bacterial challenge in non-ventilated lungs, as expected, induced an increase in transcript expression of T_H_1 cytokines (*IFNγ*, *TNFα*, *IL-6*, and *IL-1β*) compared to the non-infectious control group (*p* < 0.001 for all; Figure 2B). However, for T_H_1 cytokines, the MV+P_A_ group showed a reduction only in the transcript levels of *IFN-γ* compared to the P_A_ group (three-fold, *p* < 0.05), although declining trends for *TNF-α*, *IL-6*, and *IL-1β* were also noted (Figure 2C). These data suggest that MV leads to a dampening of pro-inflammatory response, especially of the T_H_17 cytokines, after the bacterial challenge.

### 2.3. Dampened IL-17A Response in Patients with Ventilator-Associated Pneumonia

**Figure 3:**
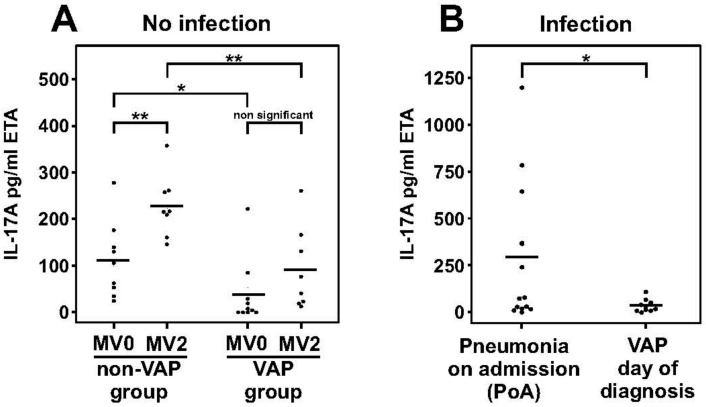
Dampened IL-17A response in ventilator-associated pneumonia (VAP) patients. (**A**) MV-induced IL-17 protein secretion in endotracheal aspirate (ETA) samples in patients who developed VAP (VAP group) and those that did not (non-VAP group) after 48 h (MV2) compared to the pre-MV timepoint (MV0). The VAP group had lower IL-17A levels than the non-VAP group at both the MV0 and MV2 timepoints. (**B**) Dampened IL-17A response was observed in VAP patients on the day of diagnosis compared to patients who arrived in the ICU with pneumonia (PoA). Line represents the average, dots are individual values of each patient; * *p* < 0.05, and ** *p* < 0.01).

We further studied the impact of MV and of VAP on IL-17 levels in endotracheal aspirate (ETA) samples from mechanically ventilated patients. ETA has an advantage over BAL in allowing an easier and frequent longitudinal sampling from MV patients. Comparing IL-17 levels in ETA samples taken from 19 patients at the start of mechanical ventilation (MV0 time point; 72 pg/mL ± 8.7 standard error of mean (SEM)), we found an increase in IL-17A protein levels after 2 days of MV (MV2 time point; 159 pg/mL ± 10.8 SEM; paired *t* test *p* < 0.001). Furthermore, 10 of these patients developed VAP, defined as development of pneumonia after 48 h of mechanical ventilation. Stratifying the patients into VAP (*n* = 10) and non-VAP (*n* = 9) groups, we found that IL-17A levels in the VAP group were lower at the start of mechanical ventilation (MV0) compared to the non-VAP group (37 pg/mL ± 13.9 SEM versus 112 pg/mL ± 17.9, respectively; *p* = 0.044). After two days of mechanical ventilation (MV2), patients who developed VAP continued to show a significantly lower level of IL-17A compared to patients who did not develop VAP (91 pg/mL ± 22.0 SEM versus 227 pg/mL ± 16.6 SEM, respectively; *p* = 0.003; Figure 3A). The Acute Physiology and Chronic Health Evaluation (APACHE) II scores, a validated measure of severity of critical illness [29], in the VAP and non-VAP groups did not significantly differ (23.4 ± 18 SEM versus 24.8 ± 1.5 SEM, respectively; *p* = 0.699). In earlier studies neutropenia has been shown to be associated with increased risk of infection and lymphopenia has been linked with development of septic shock [30,31]. However, no significant differences between the VAP and non-VAP groups existed for total white blood cell count at MV0 (12.8 ± 3.34 10E9/L versus 15.3 ± 2.45 10E9/L, respectively; *p* = 0.552) and at a MV2 timepoint (15.5 ± 1.72 10E9/L versus 13.6 ± 1.25 10E9/L, respectively; *p* = 0.606). Differential data was also available for all patients for the MV0 timepoint and did not show significant differences between VAP and non-VAP groups for either neutrophils (10.0 ± 0.45 10E9/L versus 13.0 ± 2.19 10E9/L, respectively; *p* = 0.465) or lymphocytes (1.5 ± 0.22 10E9/L versus 1.3 ± 0.23 10E9/L, respectively; *p* = 0.682). These clinical data suggest that MV induces a significant increase in IL-17A levels in mechanically ventilated patients and that patients on MV having low levels of IL-17A are associated with a higher likelihood of developing VAP compared to patients on MV with on average higher levels of IL-17A.

To understand if MV had an influence on IL-17 levels in VAP patients versus those who develop pneumonia without mechanical ventilation, we compared the VAP patient cohort (*n* = 10) with 13 randomly selected patients who were admitted to the ICU with pre-existing pneumonia (pneumonia on ICU admission; PoA). The PoA patients had an APACHE II score of 33 ± 13.5 SEM, which was not significantly different from that of the VAP patient cohort (23.4 ± 18 SEM; *p* = 0.106). Although samples (ETA) from PoA patients were taken after MV was initiated, the duration of MV here (2 ± 0.9 SEM MV days) was significantly shorter than the duration of MV before patients developed VAP (4.9 ± 2.8 SEM MV days; *p* < 0.001). Examining IL-17 levels in VAP patients at the day of VAP development (VAP0 timepoint), we showed that IL-17 levels were nine-fold lower in the VAP group (32 pg/mL ± 6.7 SEM; *n* = 10) compared to that in the PoA group (292 pg/mL ± 74.8 SEM; *n* = 13; *p* = 0.041; Figure 3B). Moreover, the total white blood cell count did not differ between the VAP and PoA groups (*p* = 0.703; see Table 1), and while neutropenia was an exclusion criterion for the enrolment of VAP patients, one patient was neutropenic in the PoA group. Excluding the latter patient from our analysis did not alter the significant differences observed between the VAP and PoA group (*p* = 0.041). These data suggest that like rodents, IL-17A levels were also dampened in patients with VAP in comparison with non-ventilator associated pneumonia.

## 3. Discussion

VAP is estimated to occur in 9–27% of all mechanically ventilated patients and contributes to approximately half of all cases of hospital-acquired pneumonia [2,32]. Approximately 50% of all antibiotics administered in ICUs are for treatment of VAP [32]. Improved general management of ventilated patients has somewhat reduced VAP incidence in the last decades, however, the mortality and morbidity associated with VAP has not abated. This is because while there are several risk factors associated with VAP development and pathogenesis, the precise mechanism leading to VAP remains unknown.

MV, obviously the biggest risk factor for VAP, has been demonstrated to cause induction of proinflammatory cytokine expression and innate cell infiltration in a tidal volume-dependent manner [7,9,10,11,12,13,14]. However, proinflammatory cytokines are anti-bacterial and per se do not explain VAP development. Using a rodent VAP model, we recently showed that MV-induced lung inflammation caused elevated IL-4 secretion and M2 macrophage polarization with reduced bacterial phagocytic capacity [7]. This suggested that besides the classically studied T_H_1 cytokines, other immune pathways are involved in ventilation-induced lung inflammation and in VAP. One of the important functions of IL-4 is in maintaining the balance between proinflammatory and anti-inflammatory pathways by suppressing T_H_1 and T_H_17 cytokines [10].

Several studies have shown the importance of IL-17 in various physiological and pathophysiological processes, including host defense against infections, especially to Gram negative and fungal infections [11,13,33,34,35,36]. For instance, mice treated with anti-IL-17A neutralizing antibody showed a reduced bacterial clearance in a *P. aeruginosa* lung infection model [33]. Similarly, IL-17A overexpression led to increased survival of animals and decreased bacterial burden in Gram-negative pathogen infection models [11,34]. Furthermore, a murine vaccination experiment against *P. aeruginosa* showed a protective effect due to an increased IL-17 response upon infection [37]. Importantly, hospital-acquired infections with Gram-negative pathogens are a major current challenge in hospitals [38] and many of the VAP-causing Gram-negative pathogens are on the WHO priority list [26]. However, the role of IL-17 cytokine family in VAP and in MV, hitherto, remained largely unknown.

While IL-17A is one of the best studied and most important IL-17 cytokines, another important member of the IL-17/IL-10 family is IL-22. Increasing evidence suggests that together with IL-17, IL-22 is also a key regulator of homeostasis and epithelial barrier function and combats infections [11,13,33,34]. Concerning the cellular sources of IL-17 and IL-22, early studies suggested that both cytokines were almost exclusively co-expressed by T_H_17 cells, though recent data suggest that IL-17 and IL-22 may also be expressed by natural killer T cells (NKT cells), γδ T cells, and innate lymphoid cells; and interestingly, these cells produce IL-17 more rapidly than do T cells and are especially important in the early response to infection [12,35,36,39,40,41].

Here, we showed that MV in healthy rats activates IL-17A and IL-22 in a V_T_-dependent manner. This was confirmed by clinical data in MV patients, where two days of mechanical ventilation, in the absence of any infection, led to a significant increase in IL-17A levels in ETA, compared to the pre-ventilation timepoint levels. We also demonstrated an increase in IL-17F, the second-most important member of the IL-17 family, however, this was only noted in rodents. In patients, only three showed IL-17F above the lower limit of detection and these were amongst the group of patients with the highest IL-17A levels (data not shown). While the impact of MV on IL-17 cytokines has not been studied in humans, a study in mice addressing the impact of a high-fat diet on MV-induced inflammation reported that one hour of V_T_ 7 mL/kg ventilation led to increased IL-22 but decreased IL-17 levels [17]. However, the tissue was analyzed 24 h after the end of MV, which may have shown a healing response rather than a direct MV-induced response. Thus, our data suggest that MV-induced lung injury proficiently induces proinflammatory T_H_17 cytokines, as has been shown previously for T_H_1 cytokines [5,6,7,8,42].

Interestingly, as indicated earlier, we also previously showed that MV co-induces a significant T_H_2 cytokine response [7], and mechanically ventilated rat and mouse when followed by *Pseudomonas aeruginosa* inoculation had worsened disease progression and reduced bacterial clearance from lungs [7]. When IL-4 signaling was blocked in IL-4R knockout mice, the worsened phenotype and increased lung bacterial count observed in rodent VAP models was restored, compared to animals that received the same bacterial dose without prior MV [7,10]. Thus, to further understand how the interplay between T_H_1/T_H_2/T_H_17 cytokines impacts the development of VAP, we studied these cytokines in VAP animals that received MV followed by intratracheal instillation of *P. aeruginosa*. Spontaneously breathing animals that either received *P. aeruginosa* or remained non-infected were used as control. In accordance with data that T_H_1 as well as IL-17A and IL-22 are drastically increased in several lung and other infection models [11,33,34], we first showed that bacterial challenge with *P. aeruginosa* of lungs in non-ventilated rats induced a significant increase in T_H_17 cytokines (IL-17A, IL-17F, and IL-22) and T_H_1 cytokines (IFNγ, TNFα, IL-6, and IL-1β) compared to the non-infected/non-ventilated control group. However, the same bacterial challenge in mechanically ventilated rats (i.e., VAP rats) led to a significantly lower response for all three IL-17 family cytokines studied (IL-17A, IL-17F, and IL-22) as well as for IFNγ, compared to the infected/non-ventilated control group, although declining trends for IL-6 and IL-1β were also observed. These data suggest that MV leads to dampening of T_H_17 cytokines after a bacterial challenge in mouse models.

To study whether a similar dampening of the IL-17 response to infection exists in VAP patients, we studied IL-17A in VAP patients on the day of VAP diagnosis compared to patients that were admitted to the ICU with pre-existing pneumonia. Following clinical protocols, the latter group of patients were also started on MV, though the duration of MV in these patients was on average 2.5 days shorter than the VAP patients at the time of ETA collection. Correcting for co-morbidities and the APACHE II scores, and despite the small number of patients in this study, we showed that VAP patients had significantly lower IL-17A levels compared to non-VAP pneumonia patients. Interestingly, a reduction in T_H_17 cells in VAP patients in comparison to ventilated patients who did not develop pneumonia has been reported in one study [16]. Another study found the opposite [15]. While the precise reasons for this discrepancy is unknown, in acute infections and therefore in absence of an adaptive immune response, the numbers of T_H_ cells should not be expected to change. Recent data also suggest that in response to infection, innate cell sources are more rapid and robust in secreting IL-17 compared to T-cell sources [12,35,36,39,40,41]. Nevertheless, it remains possible that prior adaptive or varied innate cell responses can predispose patients with low levels of IL-17 to develop VAP. In support of this premise, we showed here that, corrected for APACHE II scores (co-morbidities and other covariates), mechanically ventilated patients who eventually developed VAP had significantly lower levels of IL-17A levels in their ETA samples at start of mechanical ventilation compared to those who did not develop VAP. Even after 2 days of MV, IL-17A levels in VAP patients remained significantly lower than in the MV patient group that did not develop VAP, suggesting that low IL-17 levels could be co-contributing to VAP development. These observations are, of course, based on data from a small number of patients and await confirmation in a larger patient population.

Lastly, one of the ways T_H_17 cytokines combat bacterial infection is by recruiting neutrophils and monocytes to the site of inflammation/infection [12,14,43]. Interestingly, neutrophils themselves have been identified as a source of IL-17 in tissue inflammation and infection [23,41,43,44]. While neutrophil recruitment is also regulated by several chemokines like IL-8, MMP8, MMP9, CXCL1, CXCL2, CXCL5, LTB4 etc., here we show that lung neutrophils in ventilated lungs correlate well with transcript levels of *IL-8*, *IL-17A*, and *IL-22*, suggesting that IL-17 cytokines might have important downstream effector functions in MV-related inflammation [14,21].

To conclude, our translational studies show a distinct involvement of IL-17 associated cytokines during acute MV-induced lung inflammation. Ventilation–volume dependent IL-17A upregulation also associated strongly with neutrophil infiltration in ventilated lungs, a function fulfilled in part by IL-17A, IL-22, and IL-8. Lastly, in concordance with an MV-mediated T_H_2 response shown in earlier studies [7,16], we showed that MV is associated with a dampened T_H_17 cytokine response in lungs of infected animals as well as in VAP patients suggesting that this immune incompetence could be one of the mechanisms associated with VAP development.

## 4. Materials and Methods

### 4.1. Rat Mechanical Ventilation Model

Animal experiments were conducted according to the guidelines of the Federation of European Laboratory Animal Science Associations and approved by the Ethical Commission for Animal Experimentation of the University of Antwerp (ECD 2010-42 approval date 17/01/2011). Wistar rats, aged 8–10 weeks, weight approximately 320 g, were obtained from Charles River Laboratories (Brussels, Belgium). Up to four rats were simultaneously ventilated with a Servo 900 C ventilator (Siemens, Solna, Sweden) [7]. Briefly, anesthesia was induced by a combination of 100 mg/kg ketamine and 0.5 mg/kg medetomidine. Rats were orotracheally intubated with 14 G angiocatheter (BD–Life Sciences, Ermebodegem, Belgium). Three ventilation protocols were utilized: (i) Ventilation with V_T_ 5 mL/kg (*n* = 8) for 2 h with 7 cm H_2_O peak-inspiratory pressure (PIP), 4 cm H_2_O positive end-expiratory pressure (PEEP) and 80 breaths/min, time inspiratory/expiratory (TI/E) 1:2, and 21% inspired oxygen, (ii) ventilation with V_T_ 8 mL/kg (*n* = 8) for 2 h with 10 cm H_2_O PIP, 4 cm H_2_O PEEP and 60 breaths/min, and (iii) ventilation with V_T_ approximately 25 mL/kg (*n* = 6) for 2 h with zero PEEP, 26 cm H_2_O PIP, and 40 breaths/min. Animals were continuously monitored for oxygen saturation (pulse oximetry, MouseSTAT, Kent Scientific, Torrington, CT, USA) and body temperature was maintained at 37 °C (RightTemp, Kent Scientific). After ventilation, animals were euthanized and studied. Spontaneously breathing, non-manipulated animals (*n* = 10) served as controls.

### 4.2. Induction of Bacterial Pneumonia in Ventilated Rats

For the MV+P_A_ group, animals (*n* = 8) ventilated with V_T_ 8 mL/kg for 2 h received an intratracheal dose of 2 × 10^7^ colony forming units of *P. aeruginosa* ATCC-27853 strain suspended in 500 μL saline immediately after ventilation. As controls, animals (*n* = 8) received identical bacterial instillation without prior ventilation (P_A_ group). Anesthesia was reversed using 300 μg/kg atipamezole and animals were monitored for clinical signs of pneumonia [7,45] until 24 h post-infection when they were euthanized, tissue collected, and analyzed.

### 4.3. Patient Samples

VAP and non-pneumonia patients’ samples were obtained from ‘Identification of predictive biomarkers of pneumonia in artificially ventilated patients’ (IBIVAP) study conducted at the Intensive Care Unit of the Antwerp University Hospital. Written consent was obtained from the closest relatives of patients and the study was approved by the Ethics Committee UZA (ECD: 12/12/112, approval date 07/05/2012). The exclusion criteria for enrolment were: Infection other than VAP, prior gastric or esophageal surgery, neutropenia, and expected short duration of ventilation such as after cardiac surgery. VAP was clinically diagnosed in patients ventilated for >48 h, and was based on a new or progressive consolidation on chest radiology and at least two of the following variables: Fever greater than 38 °C, leukocytosis or leukopenia, and purulent secretions [2]. At this point, respiratory samples were taken (endotracheal aspirates obtained through tracheal aspiration or bronchoalveolar lavage (BAL) and/or BAL fluid) and these were cultured to isolate the causative pathogen. A VAP diagnosis was only made when pathogens were cultured and identified in these samples. In total 100 patients were enrolled in the study of which 10 developed VAP of different etiologies. From 90 non-VAP patients, 10 patients who did not develop any form of infection were randomly selected to be studied as a control group. One patient had sepsis and was excluded from further analysis.

Pneumonia on admission (PoA) patients’ samples were obtained from ‘The Advanced understanding of *Staphylococcus aureus* and *Pseudomonas aeruginosa* Infections in Europe–Intensive Care Units’ (ASPIRE-ICU) study (NCT02413242—ClinicalTrials.gov) [46]. Although the study focuses on ICU-VAP, in this sub-study, a random selection was made of patients that arrived in the ICU with a pre-existing pneumonia (*n* = 13).

For both IBIVAP and ASPIRE-ICU studies, endotracheal aspirate (ETA) was collected in a standardized manner with a mucus extractor, transported on 4 °C and stored at −80 °C until they were batch processed as described below. IBIVAP samples were collected at the start of mechanical ventilation, on day two of MV and on the first day of VAP diagnosis. PoA patients entered the ICU with a positive diagnosis of pneumonia and were sampled after MV started. Patient details are listed in Table 1.

### 4.4. Transcript Studies

Complete right rat lung was snap frozen in liquid nitrogen and preserved for RNA extraction. The tissue was homogenized by crushing in liquid nitrogen and RNA was extracted using RNeasy-mini spin columns (Qiagen, Antwerp, Belgium). Integrity and concentrations were estimated using RNA-nanochips on Bio-analyzer (Agilent, Leuven, Belgium). RNA was converted to cDNA (RT2 First Strand Kit, Qiagen, Antwerp, Belgium) and quantitative PCR was performed on Bio-Rad CFX Connect with SsoAdvanced SYBR green supermix (Bio-Rad, Temse, Belgium) utilizing 2 step PCR with cycles of 95 °C for 10 s followed by 60 °C for 30 s. Primer sequences are shown in Table A1. Data were analyzed using the comparative CT method with *Actb* and *Sdha* as housekeeping genes as described earlier [7,47,48].

### 4.5. Neutrophil Quantification

Paraffin-embedded sections from the left lung were prepared and stained with H/E and anti-neutrophil immunohistochemistry (1:10,000, LifeSpan Biosciences LC-348181, Seattle, WA, USA). Number of neutrophils for each group was manually counted on 10 consecutive fields of 200× magnification, as described by us previously [7,25].

### 4.6. IL-17 Immunoassay

Broncho-alveolar lavage fluid (BALF) samples from rats were collected by gentle injection of 32 mL/kg body weight of cold sterile PBS in the lungs and withdrawal after 10 s of 70% of the injected solution. An IL-17A sandwich ELISA was performed on this BALF according to the instructions of the manufacturer (R&D systems, Abingdon, UK). In short, BALF samples were diluted 1:1 in assay diluent and added to pre-coated wells. Samples were incubated with a biotin conjugated anti-IL-17A antibody and subsequently with a streptavidin-labeled horse radish peroxidase (HRP). Tetramethylbenzidine (TMB) substrate was used to quantify the amount of bound protein by measuring the colorimetric reaction at 450 nm.

Patient ETA samples were mechanically disrupted by blending (30,000 rpm, probe size 8 mm) and diluted 1:1 *v/v* with Sputolysin (Boehringer Ingelheim, Ingelheim am Rhein, Germany) and incubated for 15 min. Thereafter the mixture was centrifuged at 3000 g and the supernatants aliquoted. Supernatants were measured on an IL-17A and IL-17F MSD human U-plex assay on QuickPlex SQ 120 according to the manufacturer’s instructions (Mesoscale discovery; MSD, Rockville, MD, USA). The lower limit of detection for IL-17A and IL-17F were 5.35 pg/mL and 164 pg/mL respectively.

### 4.7. Data Analyses and Statistics

Data analyses were performed using SPSS—v.24. Transcript data are presented as average fold-differences with standard errors of the mean. ELISA data are presented as averages of each group with standard errors of the mean. MSD results from patients are represented as means with the individual data points. The Kolmogorov–Smirnov test was utilized for testing normality before testing statistical significance of differences by two-tailed independent or paired *t*-tests. Values of all significant correlations (*p* < 0.05) are given with degree of significance indicated (* *p* < 0.05, ** *p* < 0.01, and *** *p* < 0.001).

## Figures and Tables

**Table 1:** Patient details.

	MV Av ± SD *n* = 9	VAP Av ± SD *n* = 10	PoA Av ± SD *n* = 13	*p*-Value (VAP vs. PoA)
Age (years)	61.1 ± 12.2	60.2 ± 11.6	62.6 ± 9.6	0.592
Gender (% male)	63%	50%	69%	
MV before sampling (days)	0; 2	0; 2; 4.9 ± 2.8	1.9 ± 0.9	0.0018
Total ICU stay (days)	20.2 ± 11.2	24.8 ± 12.8	18.2 ± 13.4	0.243
APACHE II at admission	24.9 ± 6.6	23.4 ± 8.8	33 ± 13.5	0.106
Prognosis (% deceased)	33% (3/9)	40% (4/10)	15% (2/13)	
Total white blood cell count *	13.6 ± 1.25	13.7 ± 1.60	15.4 ± 2.69	0.703

MV = mechanical ventilation; VAP = ventilator associated pneumonia; PoA = pneumonia on admission; ICU = intensive care unit; APACHE II = Acute Physiology and Chronic Health Evaluation II; *, Average total white blood cell counts (with SEM) are shown for MV2 timepoint for patients who did not develop infection, VAP0 or day of clinical diagnosis for VAP patients and at the day of admission to the ICU for patients with non-VAP pneumonia (PoA).

## Abbreviations

ASPIRE-ICU	The Advanced understanding of *Staphylococcus aureus* and *Pseudomonas aeruginosa* Infections in Europe—Intensive Care Units
BALF	Bronchoalveolar lavage fluid
ETA	Endotracheal aspirate
IBIVAP	Identification of predictive biomarkers of pneumonia in artificially ventilated patients
MV	Mechanical ventilation
P_A_	*Pseudomonas aeruginosa*
PEEP	Positive end expiratory pressure
PIP	Positive inspiratory pressure
PoA	Pneumonia on admission
VAP	Ventilator-associated pneumonia
V_T_	Tidal volume

## Appendix A

**Table A1:** Primer sequences used for this study. *ACTB* and *SDHA* were used as housekeeping genes.

Target	Host	Fw seq	Rv seq
*Actb*	Rat	GTCGTACCACTGGCATTGTG	CTCTCAGCTGTGGTGGTGAA
*Sdha*	Rat	CTCTTTTGGACCTTGTCGTCTTT	TCTCCAGCATTTGCCTTAATCGG
*Ifn-γ*	Rat	ATTCATGAGCATCGCCAAGTTC	TGACAGCTGGTGAATCACTCTGAT
*Tnf-α*	Rat	CTTCTCATTCCTGCTCGTGG	TGATCTGAGTGTGAGGGTCTG
*IL-1β*	Rat	TGCAGGCTTCGAGATGAAC	GGGATTTTGTCGTTGCTTGTC
*IL-6*	Rat	AAGCCAGAGTCATTCAGAGC	GTCCTTAGCCACTCCTTCTG
*IL-17A*	Rat	ACTACCTCAACCGTTCCACTTCA	CTTCAGGACCAGGATCTCTTGCT
*IL-17F*	Rat	CCCGGAGACCTCTCAGAAGA	GCCCTACTTTGGGGTTCCTC
*IL-22*	Rat	TCCAGCAGCCATACATCGTC	GGCTTTGACTCCTCGGAACA
*IL-10*	Rat	GCTCTTACTGACTGGCATGAG	CGCAGCTCTAGGAGCATGTG
*IL-8*	Rat	CCCCCATGGTTCAGAAGATTG	TTGTCAGAAGCCAGCGTTCAC
